# Hypophosphatemic effect of niacin extended release in ischemic kidney disease

**DOI:** 10.17179/excli2015-537

**Published:** 2015-10-14

**Authors:** Ghazala Yasmeen, Manohar Lal Dawani, Tabassum Mahboob

**Affiliations:** 1 Department of Physiology, University of Karachi, Karachi, Pakistan; 2 Ex-Head of Department, Nephrology Unit, Jinnah Postgraduate Medical Centre (JPMC), Karachi, Pakistan; 3 Department of Biochemistry, University of Karachi, Karachi, Pakistan

**Keywords:** chronic kidney disease, hyperphosphatemia, ischemic nephropathy, niacin extended release

## Abstract

Ischemic nephropathy is an emerging cause of end stage renal disease, associated with many co-morbidities especially cardiovascular disease risk and derangement in calcium-phosphorus homeostasis resulting in hyperphosphatemia, influencing bones, a characteristic of advancing chronic kidney disease. The management of elevated serum phosphorus has been a challenge in this patient population with compromised kidney performance, as available phosphorus lowering agents possess many undesirable hazardous secondary effects and/or are very expensive. While niacin in different formulation is known to not only correct dyslipidemia but also reduce phosphorus level, but its clinical use restricted owing to side effects. The objective of present study is to evaluate such effect of niacin extended release (NER) in ischemic nephropathy. The chronic kidney disease patients fulfilling the pre-defined criteria were randomly categorized into two groups of equal size (n=60) and prescribed either atorvastatin 20 mg/day or NER 500 mg/day with the same dose of statin for four months. A control of 50 healthy characters matched was also incorporated for local reference range. Baseline and follow up phosphorus concentration was measured and means were compared using t-test at SPSS version 17 with 0.05 chosen alpha. There was no difference in the baseline levels in both groups while significant (p<0.001) hyperphosphatemia was observed in both units as compared with healthy controls. The administration of atorvastatin alone for four weeks showed an insignificant decrease in phosphorus, whereas, NER significantly reduced phosphorus (p<0.001). The mean percent change from baseline to follow up further endorsed the finding as statin alone brought -13.8 % reduction in phosphorus and NER -47 % from baseline. NER, at its lowest prescribed dose once a day was well tolerated by most of the patients and demonstrated significant goal achievement of phosphorus reduction. It is concluded that NER even at low doses in renal compromised dyslipidemic patients may be a promising approach to prevent the harmful vascular, valvular effects caused by hyperphosphatemia in addition to its principal target of HDL-C elevation.

## Introduction

Renal ischemia is an outcome of renal arteriosclerosis manifested by kidney hypo-perfusion beyond level of auto-regulation that brings significant decrease in glomerular filtration rate (GFR) by affecting the whole functional parenchyma following renal vessel occlusion (Adamczak & Wiecek, 2012[1]). The most prevailed etiological findings are hypertension, hypercholesterolemia and smoking (Yasmeen et al., 2014[37]; Alcazar et al., 2001[2]). 

In ischemic nephropathy where elevated cholesterol is one of the principal causes, the basic disease genesis mechanism is comprised of plaque formation in the renal artery producing an obstruction in the blood flow exposing the renal tissue to ischemia. The damage to functional parenchyma that impairs the kidney physiology includes atrophied tubules, collapsed glomeruli and interstitial fibrosis (Lerman and Textor, 2001[20]). 

As kidneys are one of the vital organs responsible for the maintenance of homeostasis by monitoring body fluid composition, their functional derangement is associated with many other clinically significant abnormalities like electrolyte imbalance, pH changes, ischemia, bone weakness etc. that may further favor disease development. Inadequate homeostasis of calcium-phosphorus with markedly raised levels of phosphorus are supposed to participate actively in arterial hardening, hypertension, and increased risk of vascular disease with advancing chronic renal disease (Levin et al., 2008[21]; Melamed et al., 2006[23]; Kestenbaum et al., 2005[18]; Menon et al., 2005[24]). Researches based on population observational studies recommended that though lying in the normal range, serum phosphorus levels are proportional to initial events of arteriosclerosis genesis and the subsequent vascular outcomes even when its subclinical (Onufrak et al., 2008[28], 2009[27]; Foley et al., 2009[12]; Ix et al., 2009[15]). The link between serum phosphorus levels and persistent atheriosclerotic measures in graded independent manner was recorded in a clinical trial (Tonelli et al., 2005[34]).

Hyperphosphatemia has also been known responsible for lesser outcomes and increased associated mortality in patients of compromised renal function. Therefore, in patients with chronic kidney disease, Kidney Disease Improving Global Outcomes (KDIGO) suggests reduction in enhanced concentrations of phosphorus toward normal range. The epidemiological studies are suggestive of the clinical basis to control serum phosphorus as they concluded up that hyperphosphatemia is a significant risk factor for secondary hyperparathyroidism and for cardiovascular disease (Block et al., 2004[6]). Limited dietary phosphorus intake and clearance through dialysis are mostly insufficient to control serum phosphate. Therefore, there is need to start phosphate to lessen the dietary phosphorus absorbed through gut. The recently used phosphate binders, though helpful, are not supposed suitable owing to their undesirable side effects including hypercalcemia, risk of potential toxicities (e.g., aluminum toxicity) and/or high cost (e.g. sevelamer and lanthanum carbonate) (Edalat-Nejad et al., 2012[10]). 

Calcium salts supplements, on the other side, are effective and well tolerable in controlling phosphorus in end stage renal disease, but they favor cardiovascular disease and calcification of aortic valve (Block et al., 2005[7]). Summing up, a hyperphosphatemia treatment protocol is lacking with balanced efficacy and minimized secondary hazards (Coladonato, 2005[9]; Qunibi et al., 2004[31]). Phosphorus-reducing agents aimed to control CVD risk factors associated with CKD including dyslipidemia also show preservative or synergistic advantage (Pennell et al., 2006[29]), that could also be applicable to individuals with early stages of CKD or having kidney functions in normal range. 

Niacin was first reported to decrease plasma cholesterol in 1955 (Altschul et al., 1955[3]). The major clinical application of niacin has been to raise HDL and reduce triglyceride levels. 

As niacin is converted largely to niacinamide, it also inhibits intestinal phosphate absorption. Early research experiments are suggestive that niacin intake might be beneficial as prime or adjunctive therapy to treat pronounced hyperphosphatemia, a characteristic feature of end stage renal disease (ESRD) (Sampathkumar, 2009[32]; Sampathkumar et al., 2006[33]; Cheng and Young, 2008[8]). Substantial data from human trials regarding niacin extended-release (NER) administration in different patient populations demonstrated 10 % drop in serum phosphorus from baseline values (Maccubbin et al., 2008[22]; Guyton et al., 2000[13]). These frequent clinical trial findings can be justified from the direct inhibition of active transport-mediated phosphorus absorption induced by niacin in the gut. The reports of NER hypophosphatemic effects from patients with dyslipidemia lacked the information on baseline phosphorus values (Maccubbin et al., 2008[22]; Guyton et al., 2000[13]), therefore the present study was aimed to examine whether niacin extended release lowers serum levels of phosphorus when used in combination with classic treatment atorvastatin in patients with ischemic kidney disease. 

## Methodology

### Study type 

It was hospital based, prospective, randomized, analytical research plan that was executed in collaboration with Nephrology Unit of Jinnah Post Graduate Medical Centre, Karachi, Pakistan. 

### Patient screening

Initially information from all the patients visiting the Nephrology out patient department treated for chronic kidney disease was collected. They were requested to answer a structured questionnaire inquiring about their medical history, current complaints, medication and known ischemic risk factors. The questionnaire was filled in by the same interviewer to avoid any discrepancy after explaining the purpose of information gathering, assurance of personal privacy and consent of the responder. 

### Inclusion criteria

Following primary screening, patients presented with any one of the below mentioned conditions, were suspected for the presence of ischemic renal changes. These include patients with at least 5-year diabetes mellitus and/or 10-year hypertension, cardiovascular disease, hypercholesterolemia, dyslipidemia, hypoalpha and/or on lipid lowering therapy using statins.

### Exclusion criteria

The exclusion criteria encompassed age less than 18 year or more than 70 year, previous revascularization, kidney transplantation, chronic kidney failure patients on dialysis, liver cirrhosis, hepatitis, AIDS, simultaneous presence of chronic inflammatory diseases, current use of steroidal medication, failure to complete 4-week statins washout period and HDL-C > 30 mg %.

### Diagnosis

Doppler sonography was performed as diagnostic tool. Diagnosis was confirmed through renal artery Doppler scan performed by skilled technician. The scan result was considered positive if there was turbulence before and after stenosis, maximum flow velocity > 180 cm/sec at stenosis, end-diastolic velocity > 50cm/sec, post-stenotic drop in velocity, acceleration time > 0.07 seconds and slope of systolic upstroke < 3 m/s^2^ or resistance index associated with stenosis or occlusion of the segmental arteries < 0.5 (Ng et al., 2010[26]). Though, existence or absence of ischemic tissue damage was sole decision of associated nephrology consultant based on clinical picture and Doppler result.

### Treatment design

On the basis of clinical picture and Doppler scan report, finally 135 patients were recognized as potential experimental units and were requested for consent to participate in a follow up study after explaining the purpose and satisfying their queries. Fifteen of total identified individuals did not show consent so skipped. While left over were randomly (randomization by computer generated random number sequence) distributed in two treatment groups. Group 1 was prescribed atorvastatin (20 mg/day) and group 2 NER (500 mg/day) in combination with same dose of atorvastatin (20 mg/day) for 16-weeks in a 1:1 ratio. 

Information regarding personal and family medical history, demographics, current medication, socio-economic status, physical activity, smoking and other required items were taken on a structured questionnaire at day first. 

### Sample collection and analytical procedures

Blood samples were collected from antecubital vein after 12-hour fasting and either plasma or serum was separated according to the assay requirement. All processed samples were stored in 0.5 ml small cuvettes at -80 °C unless used for biochemical estimation.

Inorganic phosphorus was estimated in non-hemolysed serum samples using research grade RANDOX, UK kit. Working reagent was composed of 28 ml blank reagent (0.36 mol/l sulphuric acid, 154 mmol/l sodium chloride) mixed with 12 ml molybdate reagent (3.5 mmol/l ammonium molybdate, 0.36 mol/l sulphuric acid, 154 mmol/l sodium chloride). Standard solution was comprised of 5 mg/dl potassium phosphate. Working reagent was allowed to react with standard and samples for 10 minutes at 25 °C in separate test tubes then absorbance was measured against reagent blank at 340 nm. Inorganic phosphorus concentration was calculated using unitary method of known concentration (standard) to unknown (sample).

Sodium and potassium were measured in serum samples using Ion Selective Electrode Beckman Coulter AU clinical Chemistry Analyzer intended for quantitative measurement of electrolytes. 

According to manufacturer's instructions, hemolysis free lithium heparin serum samples were used for estimations within 48 hours after drawing. Apparatus were calibrated prior to sample readings using ISE low to high serum standards for sodium (130-160 mEq/L), for potassium (10-100 mEq/L) respectively using the particular electrodes for both ions. Sample was prepared by adding 20 μL serum in ISE buffer triethanolamine and read directly by inserting the specific electrode. Ions concentration was measured in mEq/L.

### Ethics

The study was controlled by the directions set in Declaration of Helsinki and Good Clinical Practice standards prescribed by World Medical Association (2002[35]). The plan was approved from the institutional Board of Advanced Study and Research. 

### Statistical analysis

Collected data was tabulated and reported as Mean ± S.D. t-Test was used to compare mean values through statistical software SPSS 17^®^ at chosen p-value 0.05. Percent protection induced by drug treatments was calculated by dividing the difference of pre and post treatment with control (baseline value) and multiplying the result with 100.

## Results

54 patients taking statin while 51 with statin NER combination completed the protocol successfully for four months and assessed for final changes. The basic characteristics of CKD patients with ischemic renal changes assessed for both drug combinations are illustrated in Table 1(Tab. 1). 

The effects of NER on lipid profile and endothelial function have been discussed by same authors earlier (Yasmeen et al., 2014[36], 2015[38]) and not shown here. There was marked hyperphosphatemia (p<0.001) and hypercalcemia (p<0.01) in disease control group as compared with healthy controls, the administration of atorvastatin alone for four weeks showed a significant decrease in potassium (p<0.05), insignificant decrease in phosphorus, whereas, NER significantly reduced potassium (p<0.01), phosphorus (p<0.001) while variations in serum sodium levels remained non-significant among the groups (Table 2(Tab. 2)).

On comparison of the reduction in potassium and phosphate from respective baseline to four months follow up in the patients treatment groups either on statin alone or in combination with NER, it was found that both treatment protocols brought a decrease in potassium (p<0.05, p<0.01) respectively while in case of phosphorus a generalized decrease from baseline value was observed though only significant in statin + NER group (p<0.001) (Table 3(Tab. 3)). Thus only NER administration not statin alone produced hypophosphatemic effects in patients with compromised renal function. 

**Figure 1:**
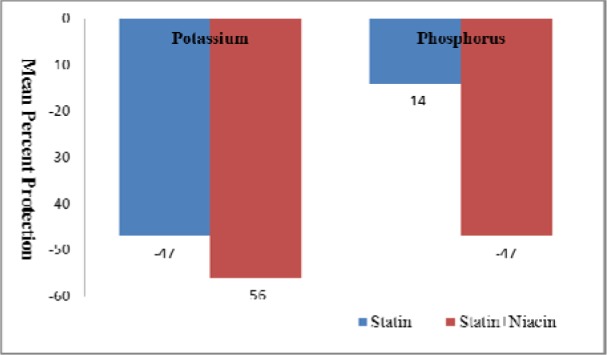
Mean percent protection induced by atorvastatin alone and atorvastatin in combination with niacin extended release in terms of potassium and inorganic phosphorus levels in patients with renovascular disease. Negative sign indicates reduction in baseline value.

The mean percent change from baseline to follow up further endorsed the finding as statin alone brought -47 % reduction in potassium as compared with NER -56 % in case of phosphorus it was observed with more difference recorded as -14 %, -47 % respectively (Figure 1(Fig. 1)). 

## Discussion

Derangments in calcium-phosphorus homeostasis particularly markedly raised serum phosphorus levels are considered to play a significant role in arterial stiffening, hypertension, and CKD associated CVD risk (Coladonato, 2005[9]; Block et al., 2005[7]). 

Recent published reports about the phosphorus-reducing effect of niacin compounds in ESRD, specially NER administered once per day, confirmed that niacin possesses the potential to lessen serum phosphorus that is similar in magnitude as attained by administration of phosphate binders agents taken three times in a day and timed requisitely to meals (Behets et al., 2004[5]; KDIGO, 2009[17]) while NER produced even better outcomes on single dose per day as we observed at low concentration of 500 mg (Table 2(Tab. 2)).

On the other hand we did not find such phosphate lowering effect when atorvastatin alone administered though it brought a reduction in serum phosphate when compared to healthy and disease control group but that was statistically non-significant (Table 2(Tab. 2)). Similarly when levels compared with follow up value with in the group, it caused 12 % decrease from baseline that was again non-significant (Table 3(Tab. 3)). To the best of our knowledge, there is no published data that reported significant statin phosphate lowering potential in compromised renal failure or in the treatment of dyslipidemia, different studies showed that its effect on phosphate is non-significant as we noticed (Amara et al., 2011[4]; Perez-Castrillon et al., 2008[30]) so focus of discussion would be NER only. 

The major problem with NER is flushing and intolerance that can be overcome by administrating the low dose initially and then buffered to high dose if required. We found the phosphorus lowering effect of NER at lower dose of 500 mg/day with a four month follow up and had significant decrease (p<0.001) in phosphorus level as compared to the baseline values (Table 3(Tab. 3)) while Muller et al. (2007[25]) used extended-release niacin with comparatively higher dose of 1000 mg/day in 20 patients for three months with similar dropout percentage as we had (15 %) because of side effects but this can be increased at high dose with large sample size. The long term use (4 vs 3 months) showed more percent reduction from baseline, i.e. 47 % (Table 3(Tab. 3)) while Muller et al. (2007[25]) reported 18 % (P<0.015) they demonstrated no significant change in serum calcium or iPTH levels.

Cheng and Young (2008[8]) used niacinamide in a randomized, double-blind, placebo-controlled crossover trial with 33 individuals on hemodialysis in dose ranging from 500-1500 mg/day and reported significant hypophosphatemic effect (P=0.02) in agreement of our results (Table 3(Tab. 3)) while no such change was observed with atorvastatin alone (Table 3(Tab. 3)). Maccubbin et al. (2008[22]) did a post hoc data analysis of phosphorus levels from 1547 patients having dyslipidemia and were randomly prescribed with extended release niacin combined with the selective prostaglandin D2 receptor subtype 1 inhibitor laropiprant (NER-L) (n=761), NER alone (n=518), or placebo (n=268). They demonstrated that NER-L and NER alone caused a sustained approximately 11 % reduction in serum phosphorus [–0.41 mg/dl (–0.46 to –0.37 mg/dl)]. The common point in all studies is that there was no significant change in serum calcium and NER showed hypophosphatemic effect at higher dose (at least 1000 mg/day) in contrast to that we administered (500 mg/day). A study done by Hyo and colleagues (2013[14]) supports our findings who reported the similar effects at same low dose of NER 500 mg/day in 31 dyslipidemic CKD patients with better tolerability. 

Animal model data highlight plausible mechanisms, especially fecal loss (Kuboyama et al., 1999[19]) responsible for phosphorus-reducing impact of niacin formulations. Around 50 % of total phosphorus absorbed in the duodenum and jejunum by an active transport pathway through the epithelial Na-Pi co-transporters contained in abundantly expressed, “ready to use” vesicles located within the small intestinal brush border (Sampathkumar, 2009[32]). The energy required for this active phosphorus transport is provided by basolateral Na-K-ATPase, Eto and fellows (2005[11]) validated in a rat model that nicotinamide impedes small intestinal Na-Pi2b expression, decreasing phosphorus assimilation and precluding the gradual elevation in serum phosphorus, a feature of kidney failure. Experimental data from healthy rats demonstrated that nicotine amide prevents sodium-dependent phosphorus co-transport in intestine, niacinamide, metabolite of niacin, prevents phosphate absorption in brush border membrane vesicles separated from rat small intestine (Katai et al., 1999[16]). The present study strength is comparatively large sample size (n=60) with clinically established chronic kidney disease of hyperlipidemic renal ischemia and longtime of administration (four months). While the study limitations are diet induced changes in phosphorus concentration and not assessed effect on serum calcium.

## Conclusion

The use of NER even at low doses in renal compromised dyslipidemic patients may be a promising approach to prevent the harmful vascular, valvular effects caused by hyperphosphatemia in addition to its principal target of HDL-C elevation. 

## Acknowledgements

The study was partially supported by a research grant from Dean, Faculty of Science’s research support program for which authors express their gratitude. 

## Conflict of interest

The authors declare that they have no conflict of interest.

## Figures and Tables

**Table 1:**
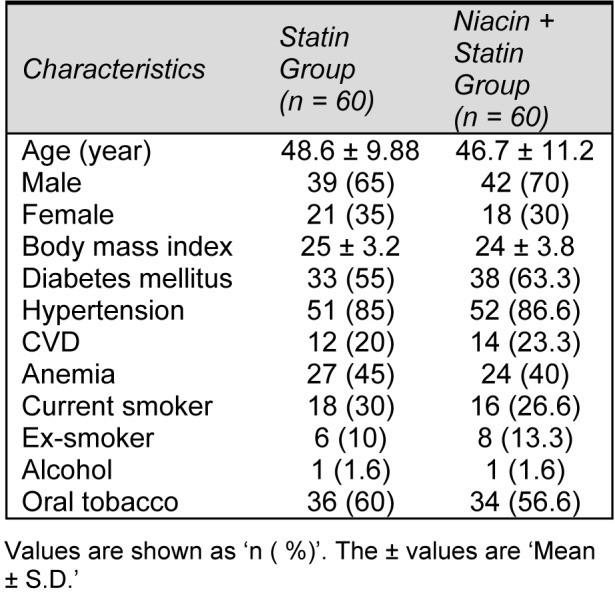
Basic characteristics of the studied patient groups with ischemic kidney disease administered atorvastatin alone or atorvastatin in combination with niacin extended release.

**Table 2:**
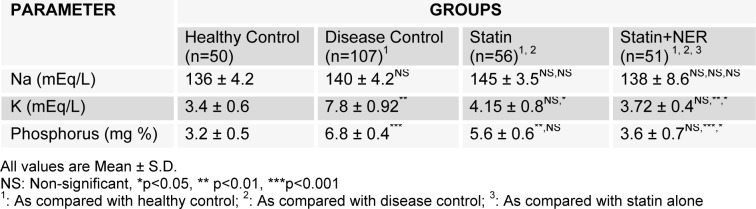
Comparison between electrolytes and inorganic phosphorus in healthy control, disease control (baseline average values of patients), patients groups of ischemic kidney disease administered atorvastatin alone and atorvastatin in combination with niacin extended release for four months.

**Table 3:**
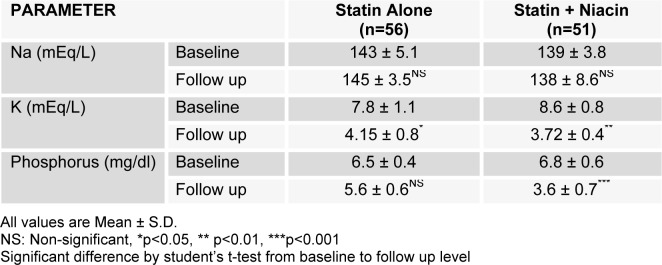
Effect of atorvastatin alone and atorvastatin in combination with niacin extended release on electrolytes and inorganic phosphorus following consumption of four months in patients with ischemic kidney disease
